# Healthcare professionals’ perspectives on minoritised ethnic young people’s access to eating disorder services in the West Midlands, United Kingdom: a qualitative study

**DOI:** 10.1186/s40337-026-01546-5

**Published:** 2026-02-18

**Authors:** Aliyah-Mae Williams-Ridgway, Sheryllin McNeil, Newman Leung, Donna Hamilton, Sukbinder Bilkhu, Anthony P. Winston, Helena Tuomainen

**Affiliations:** 1 Warwick Medical School, University of Warwick, Coventry, UK; 2 CYP Division, Forward Thinking Birmingham, Birmingham and Solihull Mental Health NHS Foundation Trust, Birmingham, UK; 3 Birmingham and Solihull Mental Health NHS Foundation Trust, Birmingham, UK; 4 Leicestershire Partnership NHS Trust, Leicester, UK; 5 Coventry and Warwickshire Partnership NHS Trust, Coventry, UK; 6 School of Psychology, University of Birmingham, Birmingham, UK

**Keywords:** Eating disorders, Population groups, Minority ethnic groups, Ethnicity, Treatment, Healthcare disparities

## Abstract

**Background:**

Minoritised ethnic individuals have comparable eating disorder rates to White populations in the United Kingdom (UK) yet face inequalities in treatment access and experiences. Understanding healthcare professional perspectives is essential for addressing these disparities. This study explores health care professional views on the access of minoritised ethnic young people to specialist eating disorder services through Levesque’s bidirectional access framework, which considers both supply and demand factors.

**Methods:**

Semi-structured qualitative interviews were conducted with 12 health care professionals from diverse personal and professional backgrounds, recruited from four National Health Service (NHS) community specialist eating disorder services in the West Midlands - the UK’s second most ethnically diverse region. Participants completed sociodemographic questionnaires and online interviews via Microsoft Teams. Transcripts were analysed using reflexive thematic analysis.

**Results:**

Health care professionals observed that young people of minoritised ethnic backgrounds accessed specialist eating disorder services less frequently than White British peers. Analysis revealed six interconnected themes spanning service-level and service-user-level factors. At the service level, systemic barriers included gatekeeping mechanisms (particularly GP referral requirements), service invisibility within minoritised ethnic communities, and inaccessible locations. Cultural and linguistic barriers were prominent, with communication challenges extending beyond language proficiency to fundamental differences in expressing distress. Health care professionals identified critical gaps in developing culturally sensitive services, citing limited resources, lack of workforce diversity, top-down organizational constraints, and insufficient cultural humility training. At the service-user level, education and awareness emerged as key barriers, with lower mental health literacy and limited eating disorder knowledge within minoritised ethnic communities as hindering recognition and help-seeking. Shame and stigma compounded these challenges, driven by eating disorder stereotypes and cultural beliefs about mental health that discouraged disclosure and treatment-seeking. Health care professionals noted that these barriers disproportionately affected minoritised ethnic individuals compared to their White British peers.

**Conclusions:**

Findings highlight multilevel barriers to accessing eating disorder services requiring comprehensive system changes including removing gatekeeping barriers, enhancing workforce diversity and cultural competency, developing targeted educational initiatives, and challenging eating disorder stereotypes. Culturally responsive services integrating these interventions are essential to improve access and outcomes for minoritised ethnic young people.

**Supplementary Information:**

The online version contains supplementary material available at 10.1186/s40337-026-01546-5.

## Abstract (plain language summary)

Eating disorders affect people from all ethnic backgrounds equally, but young people from minoritised ethnic communities in the United Kingdom face significant challenges accessing specialist eating disorder treatment services. This study interviewed 12 healthcare professionals working in eating disorder services across the West Midlands to understand these access barriers. Healthcare professionals identified problems at two levels. At the service level, barriers included requiring GP referrals to access treatment, services being unknown within minoritised ethnic communities, inconvenient locations, and language difficulties that went beyond simple translation issues. Services also lacked resources, diverse staff, and training in cultural awareness. At the community level, barriers included limited knowledge about eating disorders, difficulty recognising symptoms, and greater shame and stigma around mental health within some minoritised ethnic communities. Healthcare professionals emphasised that while accessing specialist eating disorder services is challenging for everyone, these barriers disproportionately affect minoritised ethnic young people. Improving access requires multiple changes: making services more visible and easier to access, recruiting diverse staff, providing cultural awareness training, increasing eating disorder education in minoritised ethnic communities, and addressing mental health stigma. Services must adapt to meet the cultural needs of the diverse communities they serve.

**Supplementary Information:**

The online version contains supplementary material available at 10.1186/s40337-026-01546-5.

## Introduction

Eating disorders (EDs) are complex psychiatric conditions characterised by disturbed thoughts, emotions, and behaviours around food, body image, and weight [1]. While their precise prevalence is challenging to determine, global lifetime estimates range between 0.6 and 13.1% for men and 2.6–18.6% for women [2–4]. Epidemiological data indicates that EDs affect individuals across demographic categories including age, ethnicity, socioeconomic status, gender, and sexual orientation [4–6]. Research into ED prevalence among minoritised ethnic populations shows mixed results, varying by both ethnic group and disorder type [7]. While some studies report no significant ethnic differences in ED diagnoses [8, 9], a recent scoping review found that prevalence rates among minoritised ethnic and Indigenous individuals appear comparable to, if not higher than, those of White populations in the United Kingdom (UK), Canada, Australia, and New Zealand [10]. Minoritised ethnic patients in the UK were less likely to be diagnosed with anorexia nervosa (AN) but more likely to be diagnosed with bulimia nervosa (BN), with Asian individuals generally having greater levels of disordered eating, in particular bulimic behaviours and BN [10].

Despite the severity of some eating disorders, treatment-seeking remains low, with only 30% of individuals with an ED having sought help in the UK [8] and similarly limited rates in the United States (US), where only 43% of those with binge eating disorder [BED) and BN and 33% with AN accessed treatment [11]. These already concerning figures appear worse for minoritised ethnic populations. Evidence from systematic reviews indicates minoritised ethnic individuals in the UK, US, Canada, Australia and New Zealand are less likely to seek ED treatment, receive appropriate diagnoses, be referred to specialist services, or receive treatment [10, 12]. Importantly, current prevalence data contradicts earlier assumptions that these lower referral rates reflect reduced treatment need among minoritised ethnic groups [13]. Instead, they likely result from numerous individual, cultural, and systemic barriers to help-seeking, including shame, stigma, negative cultural stereotypes and lack of clinician knowledge [10].

Several qualitative studies involving service users from South Asian communities in the UK have identified specific mechanisms through which these barriers operate. Shame and stigma around EDs and mental health, combined with lack of knowledge about EDs, consistently emerge as obstacles to help-seeking and treatment access [17, 27, 35, 40]. Additional barriers affecting minoritised ethnic individuals include poor family support, fear of mental health services, differences in how symptoms are communicated, and past negative healthcare experiences [10]. However, while barriers to *accessing* treatment have been documented, very few studies have explored what happens once minoritised ethnic individuals *enter* treatment [35]; their actual experiences of care, therapeutic relationships, and treatment processes remain largely unknown [10].

Addressing ethnic health inequalities in ED treatment requires exploring perspectives of multiple relevant parties, including those from diverse ethnic and professional backgrounds [14]. While a few UK qualitative studies have touched upon clinicians’ perspectives on ED treatment [15–17], healthcare professionals’ (HCP) views on access for minoritised ethnic groups remain largely understudied. According to Levesque’s framework, access is conceptualised as a bidirectional process influenced by factors associated with both supply (i.e., the health system and service providers) and demand (i.e., abilities of the service users and populations) [18]. As such, HCPs occupy a unique position as they witness clinical practice firsthand, deliver treatments, possess knowledge of clinical guidelines, and understand service design and implementation [19]. By interviewing these professionals, the present study adds additional valuable insights into supply-side barriers that complement existing literature, which has primarily explored access from the service user perspective.

Unlike previous research, this study focuses on minoritised ethnic young people aged 10–25 years in the West Midlands, UK—a critical period for ED onset [20, 21] and early intervention. Ethnicity is a broad construct, which is generally understood to be one’s cultural identity encompassing several factors such as ancestry, religion, language, food habits, customs, traditions and beliefs [76]. There is no global definition of ethnicity, and its conceptualisation and categorisation can vary depending upon cultural context [77]. We use the term *“minoritised ethnic groups”* to emphasise that minority status is created through social and political marginalisation, not inherent characteristics or global population size. The qualitative study aimed to understand: (1) HCPs’ perspectives on minoritised ethnic youth’s access to specialist eating disorders services (SEDS); (2) barriers and facilitators to help-seeking for EDs among this population; and (3) HCPs’ experiences working with these patients and their families.

## Methods

### Design

This qualitative study was conducted within a critical realism paradigm, acknowledging that while an independent reality exists, individual understandings are shaped by beliefs, expectations, and political, historical and social contexts [22, 23]. Semi-structured interviews were chosen to capture diverse perspectives from HCPs with varied personal and professional backgrounds. The study follows the Consolidated Criteria for Reporting Qualitative Research guidelines [24]. The study protocol was registered with Open Science Framework (https://osf.io/pkzah).

### Setting

HCPs were recruited from all four National Health Service (NHS) community SEDS in the West Midlands, UK. The West Midlands was selected as the study region due to its ethnic diversity: it is the second most ethnically diverse region in the UK behind London [25], with 23.0% of the population from a minoritised ethnic group (13.3% Asian, 4.5% Black, 3.0% mixed/multiple ethnic groups and 2.1% other ethnic group). These four services represent the complete NHS provision of SEDS in the region and covers a wide geographical area, including a mix of urban, semi-urban and rural areas. Two services catered for adults (*N* = 1 ≥ 16 years and *N* = 1 ≥ 18 years), one for all ages (> 8 years) and one for children and young people (0 to 25 years).

### Recruitment and participants

Participants were recruited via volunteer sampling between April and June 2024. The study was advertised via email to all clinicians in each service, who then reached out to researchers to express their interest. There were several recruitment attempts, and later advertisements included targeted requests for HCPs from minoritised ethnic backgrounds and specific professions (e.g., family therapists, peer support workers) based on the diversity of the sample. We aimed to recruit 12 HCPs, this being in line with Braun and Clarke’s [26] recommendation for a medium interview study, and feasible given practical constraints, including limited time and resources. Table 1 outlines the inclusion criteria for clinicians participating in interviews.

**Table 1:** Participant inclusion criteria

Criteria	Inclusion
Profession	Any healthcare professional (e.g., psychologist, nurse, dietitian, psychiatrist, support worker, family therapist)
Setting	NHS SEDS in the West Midlands
Patient group	Provide treatment to eating disorder patients aged 10–25 years and their families
Personal characteristics	Any age, sex or ethnicity
Consent	Able to provide informed consent
Language	Fluent in English

13 HCPs initially expressed interest in participating, one withdrew before completing an interview; no data from this participant were included in the analysis. This participant withdrew after being unable to complete their interview due to work commitments. A summary of demographics for the 12 HCPs who were interviewed is provided in Table 2. A range of professions were represented, including nursing staff, psychologists, therapists, psychiatrists, a service manager, occupational therapist and dietitian. Experience working in the ED field ranged from 3.5 months to 34 years (mean = 11.8 years, SD = 10.6). 

**Table 2:** Participant demographics

	Age (years)	Sex	Ethnicity	Experience working in EDs (years)	Profession
01	45–54	Woman	White British	16–20	Nurse Therapist
02	35–44	Woman	White British - Irish	16–20	Clinical Nurse Specialist
03	45–54	Woman	Asian – Indian	0–5	Speciality Doctor
04	35–44	Woman	White British	11–15	Clinical Psychologist
05	35–44	Woman	Asian – Pakistani	0–5	Senior Clinical Psychologist
06	55–64	Man	White British	31–35	Consultant Clinical Psychologist
07	45–54	Woman	White British	21–25	Consultant Psychiatrist
08	45–54	Woman	White British	16–20	Service Manager/ Clinical Lead
09	35–44	Woman	White British	0–5	Consultant Psychiatrist
10	35–44	Woman	White British	0–5	Specialist Dietitian
11	45–54	Woman	Black – Caribbean	6–10	Staff Nurse
12	18–24	Woman	Black – African	0–5	Occupational Therapist

### Data collection

Demographic data was obtained through use of a sociodemographic questionnaire (see Additional File 1). Online semi-structured interviews were conducted via Microsoft Teams.

Participants completed interviews at their home or place of work. A semi-structured interview schedule was developed by AWR and HT, with feedback provided by SM, a consultant clinical psychologist specialising in EDs (see Additional File 2). The design of the schedule was informed by Levesque’s conceptual framework of access to healthcare [18] and other similar qualitative studies (e.g. [16, 27]). Levesque’s definition of access as the ability to “identify healthcare needs, to seek healthcare services, to reach, to obtain or use healthcare services and to actually have the need for services fulfilled” ([18], p.8) was reflected in the three broad topics on which HCPs were questioned; help-seeking, access to services and treatment experience. Interviews lasted between 37 and 69 min (mean = 47.5 min, SD = 10.0). All participants were reimbursed with a £20 e-voucher.

### Data analysis

Interviews were audio recorded and transcribed using orthographic, or verbatim, transcription [26]. Transcripts were systematically coded by AWR, on NVivo [28] using a complete coding approach [26]. This approach involved systematically analysing and coding the entire dataset, rather than focusing on pre-selected segments, to ensure that all potentially relevant data were considered before developing themes. Two randomly selected interviews were independently coded by HT. Employing multiple coders in this way (i.e., collaborative coding) aimed to enhance the understanding of the data and provide more nuanced insights as opposed to reaching a consensus about each code [29].

Coded transcripts were analysed using reflective thematic analysis (TA) as described by Braun and Clarke [29]. Unlike many other qualitative analysis techniques, reflexive TA is theoretically flexible and not tied to any particular paradigm or methodology [30]. This flexibility of reflexive TA lent itself to an inductive experiential analysis in which HCPs’ perspectives and experiences of how minoritised ethnic youth access SEDS were captured and situated within social, cultural, economic and psychological contexts. Use of reflexive TA also allowed for both semantic and latent meaning to be explored, resulting in both descriptive and interpretative accounts of the data.

### Ethics

Ethical approval for this study was given by Health Research Authority (HRA) and Health and Care Research Wales (HCRW) (23/NW/0360). Written informed consent was obtained from all participants. All study data was processed and stored in accordance with the Data Protection Act 2018 and UK General Data Protection Regulation. Interview transcripts were anonymised.

Conversations around providing care to minoritised ethnic ED patients can be personal and potentially sensitive, especially when addressing perceived competence, capability, racism, or discrimination (31). The interview topic guide was carefully designed to minimise distress by using neutral, non-judgmental questions, introducing sensitive topics gradually, and allowing participants to skip questions or stop the interview at any time. The interviewer also aimed to foster a safe and non-judgemental environment, emphasising that interviews were not a test of participant knowledge. Information on available support resources were also provided to ensure that HCPs could access appropriate assistance following an interview if needed.

### Reflexivity

Interviews with HCPs were conducted by AWR, a female mixed White and Black Caribbean postgraduate researcher with psychology training and NHS SEDS experience. Reflexivity occurred throughout the study through reflective writing and supervision. Key considerations included: preventing personal experiences from overshadowing participant responses and being sensitive to ethnic/cultural variations; addressing potential social desirability bias related to the researcher’s ethnic background; and navigating power dynamics, particularly as the interviewer was younger than most participants. However, age-related power dynamics did not appear to adversely affect rapport or participant responses during the interviews.

## Results

**Fig. 1:**
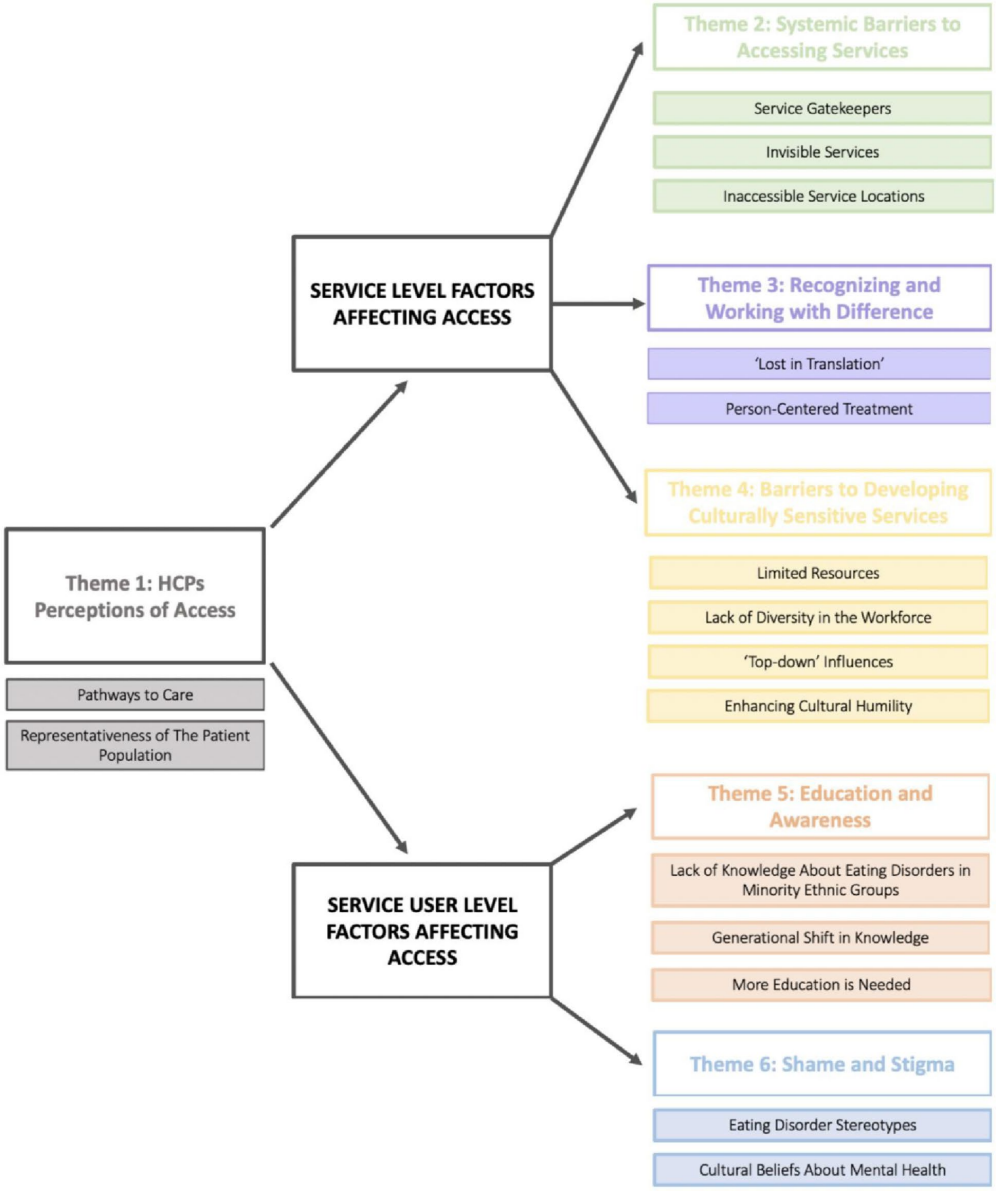
Thematic map

Major themes and relevant subthemes are outlined in Fig. 1. Themes are organised under two overarching themes: ‘Service Level Factors Affecting Access’ and ‘Service User Level Factors Affecting Access’. Quotes supporting each theme are available in Table 3.

### Theme 1: HCPs’ perceptions of access

HCPs’ perceptions of how ethnicity impacts access to ED treatment varied. Most HCPs, however, felt that minoritised ethnic youth face “significant barriers” (HCP06) to accessing treatment and are less likely to seek help or engage with SEDS compared to their White British counterparts.

#### Pathways to care

Some HCPs reported that minoritised ethnic youth delayed help-seeking until their physical health was compromised, presenting at services “really medically unwell” (HCP02) or “severely underweight” (HCP05). Other respondents discussed how for some minoritised ethnic youth identification of their ED was “coincidental” (HCP03), for example, during a routine medical appointment or a visit to an accident and emergency (A&E) department.

#### Representativeness of the patient population

Due to knowledge gaps regarding population “statistics” (HCP04) and the demographics of the local population, some HCPs felt unable to comment on the representativeness of the patient population. However, while some of the more experienced HCPs reflected that the number of minoritised ethnic patients accessing SEDS has increased, most felt that minoritised ethnic youth remain underrepresented based on the epidemiology and demographics of service catchment areas. Particularly poor access was noted for Black African, Black Caribbean and Chinese communities. Boys and men were also thought to be significantly underrepresented. Conversely, some HCPs felt that South Asian patients and minoritised ethnic university students were better represented amongst the patient population.

### Service level factors affecting access

### Theme 2: systemic barriers to accessing services

HCPs expressed that the design and set up of SEDS made access challenging for all individuals. However, it was suggested that this “general inaccessibility” (i.e., barriers affecting all patients) may be “exacerbated” (HCP06) for minoritised ethnic groups, with certain aspects of service design disproportionately disadvantaging those from diverse ethnic, cultural and linguistic backgrounds. These are linked to service gatekeepers, the invisibility of services, and inaccessible service locations.

#### Service gatekeepers

HCPs identified General Practitioners (GPs) and mental health services as ‘gatekeepers’ to SEDS, noting concerns around primary care inaccessibility and difficulties in obtaining a GP referral. Several HCPs felt that patients are often “dismissed” (HCP10) by GPs and must convince them of symptom severity to be referred. Several factors - including limited self-assurance, poor English language skills, and misconceptions about who EDs affect - were thought to make this particularly challenging for some minoritised ethnic individuals. HCPs also thought that willingness to engage with primary care differed by ethnicity. For example, concerns around confidentiality between GPs and family were identified as a barrier to help-seeking for South Asian groups specifically, potentially reflecting closer family and community networks where patients may share healthcare providers with relatives.

Likelihood of a referral being made and accepted by SEDS was also thought to vary by diagnosis or clinical presentation. Some HCPs felt underweight patients or those with AN are more likely to be referred than those with a binge-type ED or avoidant/restrictive food intake disorder (ARFID). This disparity was attributed to the greater symptom visibility and perceived severity in certain diagnoses, as well specific referral criteria (e.g., weight thresholds) or commissioning arrangements limiting some SEDS to particular ED diagnoses. HCPs advocated for more “comprehensive” (HCP07) services accepting wider presentations and engaging in earlier intervention. Increased flexibility in referral routes (e.g., accepting self-referrals) was also recommended.

#### Invisible services

Several HCPs mentioned that SEDS frequently operate with limited “visibility” (HCP09). This lack of awareness extends not only to the general public but sometimes referring clinicians themselves, who may be uninformed about the existence of these services and the specific treatments they provide.

#### Inaccessible service locations

The geographical location of some SEDS was also considered to be a barrier to access. Some HCPs identified that certain services are poorly serviced by public transport, presenting a challenge for patients without the financial means to afford car ownership or taxi services, with HCPs suggesting that this financial barrier may disproportionately affect young people from minoritised ethnic backgrounds.

Paradoxically, other HCPs felt some services were located too close to home, which could increase concerns around confidentiality for individuals who fear being recognised while attending treatment, with HCPs suggesting this may be particularly heightened for young people from minoritised ethnic communities due to perceived mental health stigma and tight-knit community networks.

### Theme 3: recognising and working with difference

All HCPs agreed that SEDS should continually strive to be culturally sensitive and informed. They emphasised that SEDS need to develop and maintain awareness of diverse cultural health beliefs and practices, while also understanding how EDs might develop and present across various ethnic groups. This cultural responsiveness should occur from initial assessment through to discharge.

#### Lost in translation

HCPs identified miscommunication between service users and providers as a significant barrier to ED treatment access and engagement. Language differences were frequently cited, with some HCPs suggesting language proficiency impacts access more significantly than ethnicity itself. Limited English fluency creates additional difficulties in navigating healthcare systems and participating effectively in therapy. Important nuances may be lost during communication. While interpreters and translated resources (e.g., service leaflets, self-help guides, psychoeducational videos) were deemed necessary, HCPs reported numerous challenges when working with interpreters, ranging from logistical difficulties to more substantive concerns, such as “increased dropout rates” (HCP08) and reduced quality and effectiveness of therapy.

Beyond language, cultural beliefs also contributed to miscommunication. HCPs noted how some communities may lack a “language for mental health” (HCP06), with some minoritised ethnic individuals, especially those of South Asian descent, presenting with “physical symptoms or somatisation as opposed to verbalising thoughts and feelings” (HCP07). Cultural attitudes regarding authority were noted to influence communication, preventing patients from “challenging professionals” (HCP05) and “putt(ing) people out” (HCP02), potentially leaving needs unaddressed.

HCPs emphasised the importance of recognising unconscious biases and avoiding assumptions about patients based on ethnicity, religion, or culture. Problematic assumptions had been made in relation to food preferences, cultural foods and eating practices, religious observances (e.g. clothing choices, fasting), ED aetiology and patients’ conceptualisation of their condition. HCPs stressed the need to avoid applying Eurocentric norms and ensuring cultural or religious preferences are not pathologised or misconstrued as disordered eating. Such assumptions were seen to lead to miscommunication, impeding the quality of care and fracturing the therapeutic relationship, leading to disengagement. One HCP noted when treatment approaches conflict with cultural or religious beliefs, families may feel as though they “aren’t heard” or that services work against them. This could make it difficult to “get them on board”, reinforcing the idea that ED services are “not for [them].” (HCP05).

#### Person-centred care

Despite occasional competing or conflicting beliefs between young people (and their family members) and services, HCPs suggested that a collaborative approach to treatment could accommodate both perspectives. Young people should be at the centre of treatment and take an active role in developing their care plan. HCPs also described how ED treatment needs to be flexible and tailored to the needs of an individual, their family and “their narrative, and how they understand eating disorders” (HCP05), to ensure they feel understood and enhance engagement.

### Theme 4: barriers to developing culturally sensitive services

HCPs were keen to see change to improve the accessibility of SEDS for minoritised ethnic individuals, however, they identified several barriers that currently hinder positive change within services.

#### Limited resources

HCPs identified service improvement efforts as challenging due to limited time, funding and resources. A particular concern was the lack of qualified staff. Some HCPs reported that due to current service demands and “small numbers of staff” (HCP06), SEDS were already “on [their] knees with capacity” (HCP08), with some services having to reduce support for family and friends, increasing group work, and not being able to provide early intervention services. Some HCPs expressed doubts about their ability to undertake and implement quality improvement initiatives and were concerned services would not have the resources to cope with increased referral numbers.

#### ‘Top-down’ influences

HCPs noted that service design and delivery is heavily influenced by local and national policies and guidelines (e.g., NICE guidelines, key performance indicators). HCPs reported feeling restricted by this, sometimes feeling that “ticking a box” was prioritised over “meeting patient needs” (HCP08). They expressed concerns that “top-down” guidelines (HCP04) may inadvertently hinder service inclusivity and accessibility. For example, while expected to deliver evidence-based treatment, HCPs reflected that most ED research focuses on White girls and women, and treatments have been “designed to be [delivered] in a particular language, embedded in a particular cultural context” (HCP06). HCPs therefore felt uncertain whether existing treatments would be as effective for those outside this demographic. Those wishing to offer more individualised or culturally adapted treatment faced a lack of evidence-based ED guidelines for working with minoritised ethnic individuals.

#### Lack of diversity in the workforce

Though workforce diversity varied across the SEDS, most HCPs felt that having a workforce representative of the community is crucial for delivering culturally sensitive treatment. A diverse team was viewed as a learning opportunity, with HCPs discussing how drawing on staff members’ lived experience and knowledge of cultural and religious traditions and practices facilitated care for diverse populations. Some HCPs felt care for non-English speakers would improve with more multilingual staff who, unlike interpreters, possess specialist ED knowledge that may enhance treatment engagement and outcomes.

HCPs had varying opinions on how clinician ethnicity may impact therapeutic alliance. Some reported that minoritised ethnic patients might feel uncomfortable working with someone from their own background due to concerns about confidentiality or judgement. Conversely, others highlighted that ethnic or cultural matching could enhance rapport and disclosure, perhaps due to perceived cultural empathy or shared experiences. The value of peer support workers, especially those from minoritised ethnic backgrounds, was emphasised, especially as a source of hope or motivation for young people.

However, HCPs stressed that improving care is not simply about ethnicity-matching. Rather, a diverse workforce increases patient choice in who they work with. First contact and impression were also considered important, with some HCPs suggesting that simply seeing someone who looks like them can be welcoming and create a feeling of safety for some patients.

#### Enhancing cultural humility

HCPs agreed that to deliver equitable, high-quality treatment, clinicians must feel confident in delivering culturally informed interventions and working with diverse populations. Although most HCPs reported feeling confident in working with patients of any background, some colleagues were recognised as uncertain about what constitutes culturally sensitive practice. For example, some clinicians may be apprehensive about using inappropriate language and unintentionally causing offense, having “difficult conversations” (HCP08) around topics such as identity or “issues that affect BME communities” (e.g., experiences of discrimination, political events), or exploring how these impact treatment and recovery (HCP05). Limited confidence in navigating language difficulties was also noted.

Confidence in working with minoritised ethnic youth and their families was thought to increase with experience. Training was perceived as beneficial in improving clinician knowledge, though its quality, quantity and content varied greatly across professional disciplines and SEDS. Some HCPs acknowledged that mastery of every culture is impossible; instead, clinicians should engage in continual learning. This requires honesty about knowledge gaps, professional curiosity, and positioning young people and their families as experts by drawing upon their lived experiences.

### Service user level factors affecting access

### Theme 5: education and awareness

HCPs associated higher levels of mental health literacy (MHL; knowledge and understanding of mental health conditions and available treatments) among young people and their families to earlier and more effective intervention. Minoritised ethnic communities, however, were generally perceived as having lower MHL. Limited knowledge and awareness of EDs was thought to be a barrier to recognising EDs and accessing SEDS.

#### Lack of knowledge about eds in minoritised ethnic groups

Several HCPs noted that compared to the “White British” population, minoritised ethnic communities often had a less comprehensive “understanding of what EDs are” (HCP05), including the warning signs and symptoms to look out for. Knowledge of healthcare systems and the appropriate help-seeking pathways was also thought to be limited.

Some HCPs spoke about how, regardless of ethnicity, the ego-syntonic nature of ED symptoms and cognitive impairment caused by malnutrition can result in individuals lacking insight into the severity of their illness and failing to recognise “how ill they are” (HCP12). Misperception of the severity of EDs and the associated physical and psychological consequences, however, was believed to be more pronounced in some minoritised ethnic groups. Several HCPs questioned the degree of “importance” (HCP03) some cultures placed on mental health and suggested that in addition to limited insight, some minoritised ethnic families or communities might not view EDs as a “valid illness” requiring treatment (HCP04). Lack of perceived severity may translate into a reduced sense of urgency to seek help and a delayed presentation to services.

#### Generational shift in knowledge

HCPs thought that the understanding of EDs has increased across generations in minoritised ethnic families. HCPs indicated that in comparison to grandparents or parents, who may have been born abroad, younger generations tend to have a greater awareness of EDs. Both school education and social media exposure were thought to play a role in this shift. Several HCPs also observed generational differences extending beyond the conceptualisation and understanding of mental health or EDs. Differences in opinions, outlooks and perspectives on a range of topics, including food choices, religious beliefs, relationships and body image were identified. First language and fluency in English also sometimes differed between young people and older family members.

HCPs viewed that generational differences in the understanding of EDs, language abilities and cultural values and beliefs had significant implications for access to and engagement in ED treatment. For example, minoritised ethnic parents may not understand the importance of or prioritise ED treatment, resulting in their child missing appointments or attending them alone with little to no family input. Generational differences could also result in conflict and communication difficulties between parents/grandparents and young people, leading to both parties feeling misunderstood or rejected, and in some cases the young person being reluctant to involve family in treatment. HCPs saw this as an important area to address in treatment.

Perceptions of the level of family involvement seemed to differ across professions. There was a pattern of responses whereby fewer ethnic differences in treatment engagement were reported for interventions targeting physiological needs. One HCP suggested that minoritised ethnic parents may struggle to “get on board” with, and understand the value of, psychological therapy (HCP05). Compared to physical health monitoring or medical procedures (e.g., ECG, blood tests, prescribing medication), psychological therapy may seem less tangible and significant. The importance of involving family was stressed by HCPs, some referring to NICE guidance (2017) which recommends family-based treatment (FBT) as the first line treatment for children and young people with AN or BN. Others felt that, regardless of age, working collaboratively with both the young person and their family led to better treatment outcomes. Where parents are absent and/or reluctant to engage in treatment it was suggested that, if appropriate and with consent, older siblings could be involved instead. The importance of not misinterpreting a limited or different parental understanding of EDs as indifference or neglect was emphasised.

#### More education is needed

Despite the perceived increase in awareness across generations, HCPs felt awareness of EDs could be further improved within minoritised ethnic communities and the wider general population to overcome stigma and encourage help-seeking, though opinions varied on optimal approaches.

HCPs highlighted various awareness strategies (e.g., national awareness campaigns, TV shows, books, social media, blogs), with social media considered particularly important among younger generations. Some advocated for SEDS to “change with the times” and leverage social media positively to advertise services and disseminate ED information (HCP02). One HCP noted that one-off events and “awareness days” only temporarily increased referrals from minoritised ethnic communities, suggesting ongoing media attention for lasting impact.

Other suggestions included SEDS developing meaningful long-term relationships with educational institutions, third-sector organisations, places of worship and other healthcare services. These connections would enable SEDS to advertise themselves and educate minoritised ethnic communities about EDs and help-seeking pathways, while simultaneously gaining further insight into different cultures and the needs of specific populations.

HCPs highlighted psychoeducation’s value (e.g., videos, leaflets, self-help guide), noting they often spent additional time educating minoritised ethnic families about EDs and treatment options due to lower MHL, which improved motivation and treatment engagement.

### Theme 6: shame and stigma

Most HCPs felt that despite progress in public perceptions of mental illness, significant stigma around EDs remains. Shame and secrecy were identified as being central to ED psychopathology and barriers to accessing SEDS for all individuals, regardless of ethnicity. However, ED stereotypes and differing spiritual, religious and cultural beliefs were thought to intensify the shame and stigma experienced by minoritised ethnic individuals.

#### Eating disorder stereotypes

HCPs spoke about how the prevailing narrative that EDs only affect “young, White, middle class females” (HCP08), is not only incorrect but also harmful. HCPs discussed how the lack of representation of EDs among minoritised ethnic populations means young people and their families often do not “identify” with their diagnosis (HCP06) or recognise EDs as an issue affecting their community. Misconceptions around needing to be a “certain weight” or have a “really low BMI” (HCP05) were also thought to delay recognition of EDs, especially for minoritised ethnic individuals who may be predisposed to having a higher or lower BMI. More generally, not fitting the mould of a typical ED patient was also assumed to be “embarrassing” and "shameful”, potentially further delaying help-seeking and disclosure of difficulties (HCP08).

#### Cultural beliefs about mental health

HCPs highlighted that there are often stigmatising views about mental illness within minoritised ethnic communities, especially South Asian populations. HCPs perceived there to be high levels of shame associated with seeking professional support and being known to mental health services, the negative connotations attached to having a mental illness extending beyond the individual to the entire family. To maintain appearances, some minoritised ethnic families may have a preference to resolve difficulties themselves, viewing mental health as a “private matter” (HCP09). Similarly, some HCPs recalled minoritised ethnic young people who concealed their ED from family and community due to fear of judgment and being misunderstood, often struggling alone in silence.

**Table 3:** Example quotations reflective of each theme

Theme	Sub-theme	Quotation
Healthcare Professionals’ Perceptions of Access	*Pathways to care*	• “I would say they come a lot sicker than perhaps some of the White British um patients do […] I feel like sometimes they leave it a lot longer to seek help.” – HCP10
	*Representativeness of the patient population*	• “I can probably count on my fingers how many people I’ve seen over the last er three years who are not White.” – HCP03 • “I think there’s been a slight increase recently, but still probably not erm as much as we’d like compared to […] the diverse population we have in [name of city].” – HCP05 • “Thinking about the client population, this is completely anecdotal, but my sense is that that the majority of ethnic minority clients that I’ve seen have come through the university not through the local population.” – HCP04
Systemic Barriers to Accessing Services	*Service gatekeepers*	• “Sometimes GPs might not necessarily pick up on eating disorders in BME groups.” – HCP05 • “You’ve got to get to your GP, and you’ve got to convince your GP you’re unwell enough to get help. Umm if you are relatively fluent in English and relatively assertive, you might get that.” – HCP06 • “Our service is very good at seeing kind of all types of eating disorders, whereas I know some areas have kind of a weight threshold because they can’t see everybody […] like some services don’t see binge eating disorder at all and we do.” – HCP01 • “I’ve worked with Asian patients who their, their GP knows everybody in the family. And so there’s not a sense of feeling that things are going to be confidential as they would like. So they don’t go to that access point to access services because they don’t want the GP to know and the whole family to know and the whole community to know.” – HCP08
	*Invisible services*	• “It’s not necessarily visibility to the public even, its visibility amongst the local health service for us because people aren’t understanding our service within the wider mental health service even.” – HCP09
	*Inaccessible service locations*	• “Because we cover the whole of the [name of region], if someone doesn’t wanna be seen in the centre that’s right by the house, I’ve got a whole heap of different places I can see you in. So it’s not really a difficult thing for us to do either.” – HCP08
Recognising and Working with Difference	*‘Lost in translation’*	• “There’s lots of things that you do moment by moment in therapy [that] get lost in translation. So we, you know, a lot of what we’re then offering is not the therapy we would like to offer, but something […] much more rigid, much more boundary, much more structured.” – HCP06 • “So if I think back to a family, um who I worked with […] mom used to often say to me she used to make a Jamaican stew […] it wasn’t until I seen the stew and realised that it was just some vegetables and some water […] I was expecting a really wholesome stew with dumplings and the lot.” – HCP02 • “Some South Asian women, erm particularly from Pakistani backgrounds or who are Muslims, might often come in and [parents] say things like erm not necessarily that she is possessed, but that somebody’s, there’s something known as the evil eye. So families might say ‘someone’s put an evil eye on her’ and we you know, ‘we’re taking her to the Mosque’ or ‘we’re taking her to see an Imam’.” – HCP05 • “Consider […] if people are covering up because of their cultural or they’re covering up for their body image, eating disorder.” – HCP01
	*Person-centred treatment*	• “That’s my opening line. We’re all working together. We’re all on the same page. We might have difference of opinions, and we might have different views, but that’s ok as well, because it’s important that […] they need that say in the treatment as well.” – HCP02 • “Identity has to be taken into account all of who we are and so it’s important that you know clinicians um feel comfortable to be able to open up discussion around things like, you know, ethnicity, the colour of people’s skin, their religion, the languages they talk, their sexuality.” – HCP08
Barriers to Developing Culturally Sensitive Services	*Limited resources*	• “I guess the dilemma with accessibility is you’ve got to have enough staff to treat the patients you’re gonna open up to.” – HCP06
	*‘Top-down’ influences*	• “We have a very White European centric system of you know research and what works and what doesn’t work […] we’ve got top down stuff coming and saying we’ve got to provide certain approaches which are all the stuff out the NICE guidelines which are you know does the research actually represent all of the different ethnic groups and how they respond to different treatment approaches.” – HCP04
	*Lack of diversity in the workforce*	• “We’ve got a very mixed demographic within our team […] and within our peer support workers which I think, it’s *really* helpful because then it’s not only kind of saying somebody can recover, but somebody can recover like me I think is probably better than any literature you can send.” – HCP01 • “It’s not so that patients have that one particular staff [member] that they can go to but it’s just giving them the option of knowing that there’s somebody with a shared experience to them and someone who maybe understands them without them even having to narrate all their life history all over again.” – HCP12 • “Obviously because I’m not the same race, I think they were a bit taken aback that I was asking questions and knew what, I knew what foods that you know potentially they were eating.” – HCP10
	*Enhancing cultural humility*	• “There is always a bit about trying to train people in different ethnic minority cultural issues. The difficulty with that is you’ve got too many to learn, and you might not see someone from a particular cultural or a subculture within that culture for ten years and you rather than thinking well I’ve done that course ten years ago, teach people to ask questions and liaise with and talk to their clients and also have other links in the community.” – HCP06 • “We don’t know it all and we don’t know it all all the time. And I think it’s really ignorant for any of us to presume we know everything about you know er different skin colours, different cultures, different ethnicities, we don’t, you know.” – HCP08 f • “There might be generic training, but then I think it would be kind of thinking about the expert by experience bit.” – HCP04
Education and Awareness	*Lack of knowledge about eating disorders in minority ethnic groups*	• “Whether there are kind of general feelings within a certain culture about whether eating disorders are a thing and whether you should seek treatment for them.” – HCP04 • “I know myself being from like an erm ethnic minority background erm you don’t tend to hear a lot about eating disorders” – HCP12 • “So there might be some people from who whose families come from different countries that aren’t you know, England erm or or the UK, who might see eating disorders as something that you know you don’t, don’t exist even.” – HCP08
	*Generational shift in knowledge*	• “So the over […] sixteens I would say generally won’t come in with family members and it would just be them and so they are very, very reluctant to get family involved […] usually it’s a case where they feel family aren’t supportive, won’t understand their needs, thinking ‘parents haven’t got time for me anyway’ um and so they’re kind of battling it on their own.” – HCP05 • “For people whose families might have come from countries where there’s been famine or starvation as well to then say ‘I am starving myself’ that’s going to have a big impact in the family […] they might be seen and viewed within their family system as being more like selfish, ‘Why are you doing this? We’ve got people starving and dying where we come from’, so and again I think we’re just adding what we know about eating disorders there can be a lot of guilt and shame present.” – HCP08 • “I have had a number of conversations with parents who often say, ‘well can’t you give medication, can’t you, can’t you give her something to make it, to make her feel hungry so that she does eat’.” – HCP05
	*More education is needed*	• “We did have um some awareness days that were that were run […] which did seem to help in the very short term increase referral rates, but it tends to be stuff that unless it’s in the media, people don’t think about it.” – HCP06 • “Having contact with kind of like local cultural organisations might be something that would be important in terms of, you know, making, building that awareness or connection with those groups as well um so that they’re aware of the service, but also that we can [pause] you know, learn more from them about perhaps their understanding of the local population and what their needs might be.” – HCP04
Shame and Stigma	*Eating disorder stereotypes*	• “It’s the barrier of nobody in our culture has an eating disorder.” – HCP01 • “If they have a higher BMI anyway because of maybe their genetic build up for example and they’re doing absolutely everything they can to lose weight, but they’re still at BMI of 20, 21, they still have an eating disorder but they, but they feel like they haven’t because they’re not a BMI of you know, 15, 14. Um and so that then feeds into this idea of you know ‘I’m not even doing this well’.” – HCP05
	*Cultural beliefs about mental health*	• “But that fear that actually in any way, if I let people know I’m coming, then that will get spread around and that will create a lot of shame and stigma for not just for the service user but also for their family.” – HCP06 • “It’s the idea that you know problems of the family stay within the family.” – HCP05 • “I think the more disembodied you are from your community, and particularly your immediate family, then the less that [stigma] becomes an issue.” – HCP06 • “So I think within the university demographic that’s become a lot easier to to seek help […] I think especially being away from home.” – P01

## Discussion

### Summary of findings

In our qualitative interview study, HCPs noted that minoritised ethnic youth accessed SEDS less frequently than their White British peers and faced significant barriers. They emphasised that while access challenges exist for all patients due to the specialised nature of ED services, certain service design elements disproportionately disadvantaged minoritised ethnic individuals. HCPs stressed the need for culturally sensitive care that recognises variations in ED manifestation, illness perception, and help-seeking behaviours across different ethnic groups. Implementation barriers identified included limited ED awareness, recognition difficulties, help-seeking knowledge gaps, and heightened stigma surrounding EDs within minoritised ethnic groups. HCPs emphasised that delivering culturally sensitive treatment requires a workforce that is representative of the community it serves.

### HCPs’ perspectives on minoritised ethnic youth’s access to SEDS

Several HCPs in this study perceived that South Asian patients may now be better represented in their caseloads, contrasting with earlier UK research documenting reduced referrals for this population [32, 33]. While these HCPs lacked access to comprehensive statistics to verify this observation, their perception aligns with the substantial South Asian population in the region [25], which includes multiple generations of residents and constitutes the majority in several wards. This multi-generational presence may distinguish South Asians from more recent migrant groups in the area. Black African and Chinese populations represent such recent migrant groups in the region [25], and their underrepresentation in ED services may reflect the healthy migrant hypothesis, which posits lower ED prevalence among migrant populations compared to established communities [34].

HCPs observed that many young people from minoritised ethnic backgrounds first seek ED treatment while at university. Leaving home may facilitate greater help-seeking by providing greater independence, separation from family oversight, and access to new healthcare providers, enabling more open discussions about sensitive health concerns without fear of family judgement or conflicting cultural expectations. These barriers have been identified in community and clinical studies involving young people from minoritised ethnic backgrounds [10]. Since most SEDS require GP referral, registering with a new practice away from home may provide crucial opportunities for candid conversations about disordered eating. However, this pattern also suggests that disordered eating or EDs in these communities may go unrecognised within family contexts, potentially contributing to delayed intervention occurring only after medical complications [16, 27, 35], which can significantly compromise recovery outcomes [36].

Delayed recognition may be particularly pronounced given the complex family dynamics that characterise ED development in minoritised ethnic communities. Research has demonstrated that family dynamics, including intergenerational conflict, significantly influence ED development in minoritised ethnic individuals [16, 42, 43]. Studies involving young people from South Asian communities have particularly emphasised how disordered eating often emerges as a coping mechanism for navigating cultural conflicts and identity challenges [17, 37–41, 43, 45]. While similar patterns occur in White British families [15], language barriers and differences in educational and social integration may intensify these challenges for minoritised ethnic families, creating environments where EDs are less likely to be recognised or discussed openly until young people gain independence through university attendance.

### Barriers and facilitators to help-seeking

On the service provider side, HCPs observed that GPs form part of the bottleneck linked to poor access to services for individuals from minoritised ethnic groups. Other studies, many involving service-seeking perspectives, have also noted that clinician biases may impede ED recognition in minoritised ethnic groups [16, 27, 46–48], with practitioners overlooking symptoms due to insensitivity to ethnic differences in presentation or misconceptions about ED demographics [16, 48]. Clinicians often prioritise medical tests to rule out physical illness before considering ED diagnoses [15], particularly problematic when patients present with physical health complaints.

Most HCPs identified language barriers as significant impediments to care access, particularly for non-native English speakers, consistent with findings from other ED studies [49, 50]. These communication challenges may also contribute to lower MHL levels by limiting access to health information and education materials [17, 27]. However, these communication challenges extend beyond simple language proficiency to encompass fundamental differences in how distress is conceptualised and expressed across cultures [51]. HCPs in other studies have also observed that South Asian communities often present ED symptoms through physical complaints (e.g., feeling sick) or attribute them to physical causes (e.g., thyroid disorder), rather than recognising them as mental health issues [15, 16, 27, 43]. This pattern reflects not only mental health stigma where psychological conditions may be associated with shame or weakness [16], but also deeper linguistic and conceptual differences in expressing psychological distress [51]: many South Asian languages lack direct equivalents for English terms used to describe specifically psychological distress. Instead, vocabulary typically associated with physical illness is employed to communicate both mental and physical suffering [51]. This linguistic reality can create diagnostic confusion unless interpreters possess cultural sensitivity and awareness of these vocabulary differences. Furthermore, individuals with limited English proficiency may resort to using English words conventionally associated with physical symptoms when attempting to communicate psychological distress, potentially leading healthcare providers to focus on somatic rather than psychological aspects of their presentation.

HCPs recognised that addressing these complex barriers requires more than translation services—it demands the development of culturally informed frameworks that integrate Western clinical practices with culturally relevant approaches to mental health [51]. This includes creating assessment terminology that balances clinical precision with cultural resonance, developing community-specific mental health education programs, and actively engaging South Asian communities to reduce stigma around psychological distress. Such comprehensive approaches could bridge linguistic and cultural gaps, ultimately facilitating earlier recognition of EDs and improving access to appropriate care for these populations.

On the service user side, despite most HCP identifying that public perceptions are shifting around mental illness, significant stigma and shame around EDs remain, which are commonly cited barriers to accessing ED treatment [52, 53]. Men and minoritised ethnic individuals often hold more stigmatising beliefs around EDs and mental illness, disproportionally deterring help-seeking [54, 55], potentially linked to media portrayals of ED as “White women illnesses” [54, p.17]. HCPs noted that shame and stigma are particularly pronounced in South Asian communities, where cultural values emphasise family harmony, social acceptance, and collective identity [43]. Concerns about the social repercussions of an ED diagnosis and the potential stigma extended to the family may drive individuals to take extreme measures to protect family honour [56]. Additionally, collectivist values often prioritise help-seeking within family networks before accessing external support [27].

### HCPs’ experiences working with minoritised ethnic patients and their families

HCPs recognised that local and national policies and guidelines (e.g., NICE guidelines, key performance indicators) constrained them to use ED diagnostic criteria, assessment tools and treatments developed primarily for young, White, cisgender girls and women, a limitation identified elsewhere [57–60]. While the UK NICE guidelines acknowledge that clinicians should exercise clinical judgement to make decisions “appropriate to the circumstances of the individual patient,” [71] they may be perceived as more prescriptive in practice compared to frameworks like the Australian ED guidelines, which explicitly embed recovery-oriented principles emphasising flexible, person-centred care delivery [72]. This perceived rigidity may contribute to clinicians’ concerns about deviating from standardised protocols, even when cultural adaptation would enhance care quality. This highlights the urgent need for policy reform that mandates the inclusion of diverse populations in treatment development and validation studies.

Bansal et al. [49, p.1] argue that the “monocultural and reductionist frameworks” of current mental healthcare models in the UK hinder culturally sensitive, person-centred care [44]. These monocultural frameworks—designed primarily around Western, White cultural perspectives—assume universal applicability of diagnostic categories and treatment approaches across all populations, while reductionist frameworks oversimplify complex cultural expressions of distress to fit predetermined Western criteria, stripping away important cultural context and meaning. Kanakam [16] found that therapists often encounter resistance when applying evidence-based models to minoritised ethnic patients, suggesting a mismatch between treatment approaches developed within these limited frameworks and diverse cultural needs of service users.

A recurring theme shared across the HCP interviews was the importance of having a diverse workforce for delivering culturally sensitive treatment. Goel et al. [61] argue that lack of diversity permeates the entire ED field, noting that structural barriers, including underrepresentation of minoritised ethnic groups in higher education pathways to clinical careers, limited role models, and workplace cultures that may not feel welcoming, have excluded certain racial and ethnic groups from the ED workforce. Consequently, it is predominantly individuals from the dominant culture who both participate in *and* conduct research, as well as receive *and* deliver treatment. While the NHS Workforce Race Equality Standard [62] advocates for inclusive recruitment, HCPs emphasised that clinician qualities like empathy, openness, and non-judgement, outweigh ethnic matching in building strong therapeutic alliances [49].

### Strengths and limitations

This is the first UK qualitative study exploring diverse HCP perspectives on minoritised ethnic youth access to SEDS in the West Midlands, addressing a gap in research that has rarely examined access from the HCP/clinician perspective [18]. The inclusion of HCPs from minoritised ethnic backgrounds (33%, *n* = 4) enabled exploration of how shared ethnic identity and cultural experiences influences perspectives on access and experiences with minoritised ethnic young people and their families. However, recruitment was limited to the West Midlands; future research should include HCPs from across the UK to explore broader perspectives and regional variations, and qualitative interviews with young people in the West Midlands to complement HCP perspectives.

The ED service where AWR previously worked served as a recruitment site, with all participants from this service being known to the researcher. Their participation may have been motivated by pre-existing relationships, including desire to support academic endeavours. While these prior relationships potentially enhanced information sharing for some participants, others may have been less forthcoming due to confidentiality concerns. It is also important to note that HCPs’ responses reflect their personal perceptions, which may have been influenced by their education, background, and cultural experiences; implicit or explicit biases were not assessed and may have shaped these perspectives.

Steps to ensure data trustworthiness included keeping a detailed analysis audit trail [63], enhancing analytical transparency and documenting perspective shifts [64], and keeping a reflexive journal. While member checking was not feasible within the study timeframe, peer debriefing through regular discussions with colleagues and feedback to site investigators assessed the feasibility of findings and promoted reflexivity regarding experiences and assumptions.

### Clinical implications

Delivering culturally sensitive treatment requires substantial time [65]; however, high clinical workloads limit opportunities for reflection and the exploration of cultural and religious factors and treatment adaptation [16, 66]. Staff shortages and funding limitations reflect broader mental health service underfunding [67] and further impede effective care.

Within this context, HCPs identified cultural competency training and cultural humility as essential for equitable ED care [59]. Cultural competence involves understanding and responding to cultural differences [69], while cultural humility is a lifelong commitment to self-reflection and learning about one’s biases and assumptions [68, 69]. UK clinical guidelines should therefore adopt more explicit recovery-oriented language that empowers clinicians to culturally adapt evidence-based interventions. Such guidance should clarify that cultural adaptation represents sound clinical judgement rather than deviation from best practice, providing a framework within which trained clinicians can confidently apply their cultural competency in practice.

Addressing inequities in access also requires targeted, system-level interventions [15, 27, 40, 43]. Culturally adapted public health campaigns may improve ED awareness in minoritised communities [70], while schools and colleges provide safe spaces for support, reducing stigma and protecting identity [49]. Increasing ED visibility and awareness within minoritised ethnic communities may challenge stereotypes and promote early service engagement.

Primary care remains central to ED identification and care coordination, yet GPs face challenges in identifying EDs among minoritised ethnic populations. Expanding GP training and refining referral guidelines is therefore essential. Future qualitative research exploring GP experiences and perspectives on ED identification could inform the development of targeted training and support earlier detection. At the same time, broadening SEDS referral pathways to include schools, universities, and community organisations also requires careful consideration While such pathways may reduce primary care access barriers for minoritised ethnic individuals, they risk bypassing GPs who remain responsible for patients’ overall medical care and may have valuable information critical to safe treatment. Developing formal collaborative networks with diverse services and organisations can facilitate ED identification in minoritised ethnic groups, build trust, and improve treatment engagement and outcomes, provided appropriate GP communication and coordination is maintained.

Beyond access, it is essential to examine the safety and appropriateness of current Eurocentric ED healthcare models for minoritised ethnic young people. Low engagement may reflect concerns about cultural relevance and service safety rather than merely a lack of awareness. Actively incorporating minoritised ethnic patient feedback into service design through co-production and systematic service evaluation processes is crucial for developing responsive and acceptable services.

Until greater diversity is achieved within the ED healthcare workforce, empowerment-based bridging approaches may help address current gaps. Models such as cultural liaison officers, peer support workers from minoritised communities, and train-the-trainer models can provide bidirectional consultative roles, support culturally appropriate knowledge dissemination, and offer cultural supervision to clinicians [73, 74].

At the level of treatment delivery, ED interventions should integrate cultural and religious considerations, including awareness of religious calendars (e.g., Ramadan), collaboration with religious leaders, and incorporation of relevant religious teachings into therapy and psychoeducation materials. Lived experience co-design is essential to ensure services are acceptable and do not cause harm, recognising that no therapy is better than harmful therapy.

Finally, to support the evaluation of service improvement and the interventions outlined above, SEDS should undertake regular audits to assess the representativeness of their patient population and identify disparities in access and treatment. At a national level, calls for a dedicated ED strategy for England [75] could address the systemic barriers identified in this study, provide a framework for standardised data collection and monitoring, and promote equitable access to ED services across diverse populations.

## Conclusions

This exploratory qualitative study examined HCPs’ perspectives on minoritised ethnic youth access to ED services in the West Midlands, UK. HCPs reported lower help-seeking behaviours among minoritised ethnic youth, with many presenting late to services or having their ED discovered incidentally. Barriers were identified at both service provider and service user levels. Recommendations include expanding referral pathways beyond traditional primary care routes, systematically incorporating minoritised ethnic patient feedback into service design, enhancing community awareness initiatives, implementing structured reflective practice, developing cross-organisational collaborations, and integrating cultural and religious considerations into treatment approaches. These findings highlight the need for culturally responsive ED services that address the unique needs of minoritised ethnic youth through workforce representation, targeted outreach, and adapted clinical practices to improve access, engagement, and outcomes for this underserved population.

## Supplementary Information

Below is the link to the electronic supplementary material.

Supplementary Material 1

Supplementary Material 2

## Data Availability

The data that support the findings of this study are not publicly available due to ethical restrictions and participant privacy concerns. The data contain information that could compromise the confidentiality of research participants.

## Declarations

### Disclaimer

The views expressed are those of the authors and not necessarily those of the NIHR or the Department of Health and Social Care.

## Abbreviations

AN: Anorexia Nervosa
BED: Binge Eating Disorder
BN: Bulimia Nervosa
ED: Eating Disorder
GP: General Practitioner
HCP: Healthcare Professional
MHL: Mental Health Literacy
NHS: National Health Service
NICE: National Institute for Health and Care Excellence
TA: Thematic Analysis
UK: United Kingdom
