# Supplementary material for: Healthcare professionals’ perspectives on minoritised ethnic young people’s access to eating disorder services in the West Midlands, United Kingdom: a qualitative study
Source: J Eat Disord. 2026 Feb 18;14:70. doi: 10.1186/s40337-026-01546-5 (PMC13020106; doi:10.1186/s40337-026-01546-5)
Supplement: Supplementary file 1 — Supplementary Material 1 [file 40337_2026_1546_MOESM1_ESM.docx]

**Healthcare professionals’ perspectives on minoritised ethnic young people’s access to eating disorder services in the West Midlands, United Kingdom: a qualitative study**

Williams-Ridgway A, McNeil S, Leung N, Hamilton D, Bilkhu S, Winston A P, Tuomainen H

**Additional File 1**

**SOCIODEMOGRAPHICS QUESTIONNAIRE**

for healthcare providers

Please highlight

**Age:**

18-24 years

25-34 years

35-44 years

45-54 years

55-64 years

65 and over

**Gender:**

Man

Woman

Non-binary

Prefer not to state

Prefer to self-describe ___________

**Self-Ascribed Ethnicity (How would you define your ethnicity?)**

**_________________________________________________**

**Ethnicity (Census):**

**Asian or Asian British**

Indian

Pakistan

Bangladeshi

Chinese

Any other Asian background

**Black, Black British, Caribbean or African**

Caribbean

African

Any other Black, Black British or Caribbean background

**Mixed or multiple ethnic groups**

White and Black Caribbean

White and Black African

White and Asian

Any other Mixed or multiple ethnic background

**White**

English, Welsh, Scottish, Northern Irish or British

Irish

Gypsy or Irish Traveller

Roma

Any other White background

**Other Ethnic Group**

Arab

Any other ethnic group

**Country of Birth (Specify)**

_____________________________________________________

**Fluency in English:**

Not Fluent

Fluent – Spoken

Fluent – Spoken and Written

Fluent – Other

**Religion**

*Identified Religion:*

Christian

Sikh

Catholic

Muslim

Hindu

Atheism

Agnostic

Spiritual

None

Other

**Do you practice this religion?**

Yes

No

**Current Job:**

Employed full-time

Employed part-time

Unemployed and currently looking for work

Unemployed and not currently looking for work

Student

Retired

Self-employed

Homemaker

Unable to work

Prefer not to state

**Job Title (Please Specify)**

**In what region of England do you live?**

East of England

South East

South West

Wales

West Midlands

Yorkshire / Humberside

I don’t live in England

East Midlands

London

North East

North West

Northern Ireland

Scotland

**How many years of experience do you have working in eating disorders?**

**_______** years

**INTERVIEW TOPIC GUIDE**

for healthcare providers

**Interview Questions & Topics:**

**Introduction** (follow up on some questions from demographic questionnaire)

- To begin could you briefly outline what experience you have working in ED services?
  - Have you worked in multiple ED services?
  - Have you held different roles within ED services?
  - How long have you worked in your current role?
  - How much experience do you have working with minority ethnic patients and their families?

**Help-Seeking**

- In your opinion are there any differences in help-seeking patterns and behaviours between ethnic groups?
  - If so, could you elaborate on these differences?
  - Why do you think these differences might occur?
- What do you perceive to be barriers for help-seeking for minority ethnic individuals? (And why?)
- What do you perceive to be facilitators for help-seeking for minority ethnic individuals (And why?)

**Accessing Treatment**

- Do you think minority ethnic groups face any difficulties accessing eating disorder services?
  - If so, what do you think these difficulties are?
- In your opinion are there any differences in the type of referrals or patterns of referrals between ethnic groups?
  - If so, could you elaborate on these differences?
  - Why do you think these differences might occur?
  - Given you work in a diverse and multicultural area do you think the ethnicity of the service users you see is reflective of this?
- Can you tell me a bit more about your experiences at the point of initial assessment with individuals and families from minority ethnic groups?

**Treatment Experience**

- Can you tell me a bit more about your experiences of delivering eating disorder treatment to individuals and families from minority ethnic groups?
  - What type of treatment do you deliver?
  - Can you tell me more about your experiences of engaging individuals and families from minority ethnic groups in [individual/group/family based] interventions?
- Have you experienced any challenges in care planning and delivering treatment to individuals from minority ethnic backgrounds?
  - If so, what were these challenges?

How, if so, did you overcome these challenges?

- How confident do you feel delivering treatment for individuals from minority ethnic backgrounds?
  - Have you received specific training?
  - What is your understanding and experiences of culturally adapted/sensitive treatment?

**Future Provision**

- If you could change anything about the treatment offered in your service for minority ethnic groups, what would it be? (And why?)
- In your opinion what do you think service providers and healthcare professionals could do to make services more accessible and improve treatment for people from different ethnic backgrounds?
